# Eisosomal proteins are essential for plant–fungal interaction of *Neurospora crassa* and the sweetgrass *Brachypodium distachyon*

**DOI:** 10.1038/s41598-026-56854-2

**Published:** 2026-06-09

**Authors:** Hannes Winter, Frank Kempken

**Affiliations:** Botanical Genetics and Molecular Biology, Botanical Institute, Christian-Albrechts-Universität, Kiel, Germany

**Keywords:** Plant–fungus interaction, Eisosomes, *Neurospora crassa*, *Brachypodium distachyon*, Microbiology, Plant sciences

## Abstract

Plant fungal associations encompass a continuum from pathogenic to mutualistic interactions that have profoundly shaped terrestrial ecosystems. The ability of fungi to colonize living plant tissues typically depends on tightly coordinated signaling and membrane organizations processes. Recently, we described a previously unrecognized endophytic interaction between the filamentous model fungus *Neurospora crassa* and the sweetgrass *Brachypodium distachyon*. Contrary to its long-standing classification as a strictly saprotrophic species, *N. crassa* was observed to invade root epidermal and cortical cells and to colonize the vascular bundles without inducing disease symptoms, indicating a stable nonpathogenic association. To identify functional elements in this interaction, we systematically screened *N. crassa* mutants lacking key signaling and membrane-associated components. Disruption of certain signaling pathways including MAP kinase and NADPH oxidase mutants did not impair plant colonization. However, deletion of *ncw*-6 or *div*-23, two proteins associated with and localized to eisosomes, completely disrupted plant root colonization of *N. crassa*. Eisosomes are plasma membrane microdomains in fungi that contain transporters and integral membrane proteins. These findings suggest an integral function of eisosomes in plant–fungal interaction of *N. crassa* and possibly other fungi as well.

**Supplementary Information:**

The online version contains supplementary material available at 10.1038/s41598-026-56854-2.

## Introduction

*Neurospora crassa* has long served as a cornerstone for eukaryotic genetic and fungal biology. Originally isolated from burned sugarcane and other post-fire habitats^1^. *N. crassa* is a filamentous ascomycete of the *Sordariaceae* family that thrives on dead plant material. Its fungal genome was among the first entirely sequenced genomes^2^ decades of genetic work - starting with the “one gene-one enzyme” experiments by Beadle and Tatum have established *Neurospora* as a model system to study gene regulation, circadian biology, cell-cell communication, morphogenesis and many more. Ecologically, *N. crassa* has been regarded as a strictly saprotrophic species, restricted to decomposing organic matter.

By contrast, *Brachypodium distachyon* (Poaceae) is a small temperate grass that has emerged as a model system for studying root architecture, cell-wall biochemistry, and plant-microbe interactions. *Brachypodium* originates from the Mediterranean and western Eurasia, it occupies disturbed and semi-arid environments and is closely related to important cereal crops like wheat and barley^3^. Its compact genome (~ 272 Mb), short generation time, and transformability have made it a widely used model plant for both pathogenic and mutualistic fungi, including *Magnaporthe oryzae*, *Fusarium spp*., and arbuscular mycorrhizal species^4^.

Plant–fungal associations have fundamentally shaped the evolution of terrestrial ecosystems by governing nutrient uptake, facilitating plant diversification, and fostering the emergence of complex biological interactions. Fungal lifestyles encompass a broad spectrum, ranging from pathogenic relationships and mutualistic symbiosis - such as mycorrhizal and endophytic associations - to saprotrophic behaviors^5,6^. Despite extensive research on signal transduction and effector secretion in pathogenic and symbiotic fungi, the cellular mechanisms that facilitate host recognition, attachment, and the ability to overcome host-derived challenges remain poorly understood^7,8^. At a focal point of this interface lies the fungal plasma membrane, an active boundary where environmental perception, nutrient exchange, and mechanical adaptation fall into place^9^. In recent years, eisosomes, stable plasma membrane furrows first described in *Saccharomyces cerevisiae*, have emerged as key spatial organizers of the membrane and their function. Eisosomes consist of cytoplasmic BAR-domain proteins such as Pil-1 A and Pil-1B, which scaffold the underlying lipid bilayer, and integral membrane proteins like Sur-7 and Nce-102, which define membrane subdomains enriched in sterols and phosphoinositides^10^. These microdomains are part of multiple processes like signaling, regulating nutrient transporter turnover, and stabilize the membrane against environmental stress^11,12^. Eisosomal components in pathogenic fungi have been linked to virulence and cell wall morphogenesis. Like in *Candida albicans*, where Δ*sur*-7 mutants exhibit disorganized membranes, defective hyphal growth, and attenuated pathogenicity^13,14^. Similar effects were observed in *Alternaria brassicicola*, the disruption of MCC/eisosome proteins impairs appressorium formation and reduces infection efficiency on plant hosts^15^. In these pathogenic fungi the eisosomal role in host interaction/infection via membrane coordination and signaling events seems evident, yet eisosomal role is nonpathogenic or mutualistic associations remains unexplored.

We uncovered a novel and unrecognized endophytic association between the filamentous fungus *Neurospora crassa* and the model grass *Brachypodium distachyon*^16^. *N. crassa* was found to accumulate within epidermal and cortical cells and colonized vascular tissues without causing visible disease symptoms. Fungal hyphae seemingly moving from cell to cell by using natural openings such as plasmodesma^16^. This unexpected interaction increases the known ecological aspects of the *N. crassa* lifecycle. To generate further knowledge about the molecular basis of this interaction, we screened a variety of *N. crassa* knock-out mutants targeting both signaling pathways (MAP kinase, NADPH oxidase) and plasma membrane-associated proteins. While the tested signaling mutants colonized roots similarly to the wild type, deletion strains for Ncw-6 and Div-23 two eisosomal-associated proteins could not be found colonizing the plant root cells. Eisosomes have been proposed in many different organisms, best described in *Saccharomyces cerevisiae* therefore there are many different descriptions for the eisosomal genes, we refer to their name based on Fungi.db.

By showing that classical communication-related proteins are dispensable in this system, we identify structural membrane effects - mediated by eisosomal proteins - as critical for root colonization. This shifts the focus from active molecular ‘crosstalk’ to the structural integrity of the fungal membrane as prerequisite for opportunistic colonization, marking eisosomes as an interesting target for future investigation into fungal lifestyle plasticity.

## Materials and methods

### Strains, growth conditions

The *Neurospora crassa* strains utilized in this study (summarized in Table 1) were obtained from the Fungal Genetics Stock Center (FGSC; Kansas City, MO, USA), the André-Fleißner Group (Technical University Braunschweig, Germany), or were generated in-house. All of the fungal cultures were maintained on Vogel´s Minimal Media (VMM; Vogel et al., 1956). For co-cultivation experiments involving *N. crassa* and *Brachypodium distachyon*, a modified VMM containing a reduced sucrose concentration (0.2%) was employed.

**Table 1:** *Neurospora crassa* strains used in this study.

Strains	Description	Origin
*ΔnoxR*	∆noxR: hph+, his3::ptef-mak-2-gfp	Fleißner Lab (Technische Universität Braunschweig)
*Δnox-2*	nox-2::hph, his3::ptef-mak-2-gfp	Fleißner Lab (Technische Universität Braunschweig)
*Δso*	∆so, matA, hygR, his3::Pccg1-H1-gfp	Fleißner Lab (Technische Universität Braunschweig)
*Δmak-2*	mak-2::hph, his3::Pccg1-H1-gfp	Fleißner Lab (Technische Universität Braunschweig)
*Δham-15*	ham-15::hph, his3:mak-1-gfp	Fleißner Lab (Technische Universität Braunschweig)
*Δpla-7*	mata, pla-7::hph, his3::Pccg1-H1-gfp	Fleißner Lab (Technische Universität Braunschweig)
*Δlsp-1*	Δlsp-1, his3::Pccg1-H1-gfp	This Study, AG Kempken (Christian-Albrechts-Universität zu Kiel)
*Δdiv-23*	Δdiv-23, his3::Pccg1-H1-gfp	This Study, AG Kempken (Christian-Albrechts-Universität zu Kiel)
*Δnce-102*	Δnce-102, his3::Pccg1-H1-gfp	This Study, AG Kempken (Christian-Albrechts-Universität zu Kiel)
*Δncw-6*	Δncw-6, his3::Pccg1-H1-gfp	This Study, AG Kempken (Christian-Albrechts-Universität zu Kiel)

### Plant material and co-cultivation

*Brachypodium distachyon* TR7 seeds were provided by the Botanical Garden of CAU in Kiel (Germany). Prior to sterilization, the lemma and palea were manually removed from the caryopses. Seeds were surface-sterilized in > 99.8% ethanol for 1 min by vigorous shaking, followed by two 8 min incubation cycles in a fresh bleach solution (50% (v/v) H_2_O, 50% (v/v) sodium hypochlorite solution, 0.05% (v/v) Triton X-100). After four subsequent washes in ddH_2_O, up to four seeds were placed onto VMM plates (0.2% sucrose). Plates were incubated at 4 °C in the dark for 48 h. Seedlings were subsequently grown in a climate chamber (22 °C, 60% humidity, 16 h light/ 8 h dark photoperiod) for 7–10 days prior to fungal inoculation.

For inoculation, macroconidia were harvested from slant tubes and suspended in liquid VMM (0.2% sucrose). Spore concentration was determined using a Thoma counting chamber, and a total of 1 × 10^5^ spores were applied to media in close proximity to the root system. Inoculated plants were maintained at 25 °C under 16 h light/ 8 h dark photoperiod.

### Genetic crosses and GFP labeling

To facilitate microscopic visualization, mutant strains (mat a) were crossed with the FGSC#9518 strain (mat A, expressing hH1-gfp) on WKM agar plates (Westergaard 4x solution, sucrose, pH 6.5). Following incubation at 25 °C for 21 days, ascospores were harvested in H_2_O and stored at 4 °C. Ascospores were activated via heat treatment (60 °C for 30–60 min) and stored onto VMM + SGF medium using warm agarose to isolate individual colonies. Each colony was screened via PCR and fluorescence microscopy. To ensure strain purity, a microconidial passage was performed before the verified strains were used for interaction assays and imaging.

### Sample fixation and sectioning

Infected root tissues were prepared for confocal laser scanning microscopy (CLSM) by fixation in a 4% (w/v) paraformaldehyde solution under vacuum (UNIVAPO 1000 H) for 15 min. Samples were washed extensively in 100 mM Tris/HCL (pH 8.0) supplemented with 0.1% (v/v) glycerol. Roots were divided into 1 cm segments and embedded in 5% (w/v) agarose. Longitudinal Sect.  (50 μm thickness) were generated using a ZEISS Hyrax V50 vibratome (Zeiss, Oberkochen, Germany) and placed in ddH_2_O for imaging.

### Microscopy

Initial screening was performed using ECLIPSE Ci epi-fluorescence microscope (Nikon, Tokyo, Japan) equipped with a GigE camera (Imaging Source, Bremen, Germany) and a GFP filter block (excitation: 480/40 nm; emission: 510 nm). Images were processed using NIS Elements D software.

High-resolution imaging was conducted with a ZEISS LSM 900 confocal microscope utilizing 5×, 10×, 20×, and 40× (oil immersion) Plan Achromat objectives and an Axiocam 305 color camera. GFP fusion constructs were exited at 488 nm, with emission detected between 500 and 550 nm. Detailed image analysis was performed using ZEN 3.2 Blue software, incorporating Airyscan mode for enhanced resolution.

## Results

To determine whether *N. crassa* is capable of interacting with living plant tissues, we co-cultivated the fungus with *B. distachyon* seedlings under sterile, nutrient-limited conditions. The *Neurospora crassa* strain FGSC9518 (his-3+::Pccg-1-hH1+-sgfp+, mat A) served as the control for all interaction assays. This strain exhibits intense green fluorescence in the nuclei due to the hH1-GFP fusion protein, which was essential for precise localization of fungal hyphae within the host tissues.

Sterile *Brachypodium* seeds were plated on VMM + S media with reduced sugar content and incubated at 4 °C for two days and then placed in the climate chamber. Germlings of seven days were inoculated with macro-conidiospores of the distinct *N. crassa* strains. The inoculated plants were cultivated under sterile conditions in a climate chamber for another seven days. Samples were then be fixed and sectioned with the vibratome into thin pieces for microscopy. Which revealed the fungus initially grew along the rhizoplane, forming a loose hyphal network around the root hairs and epidermal surfaces.

**Fig. 1:**
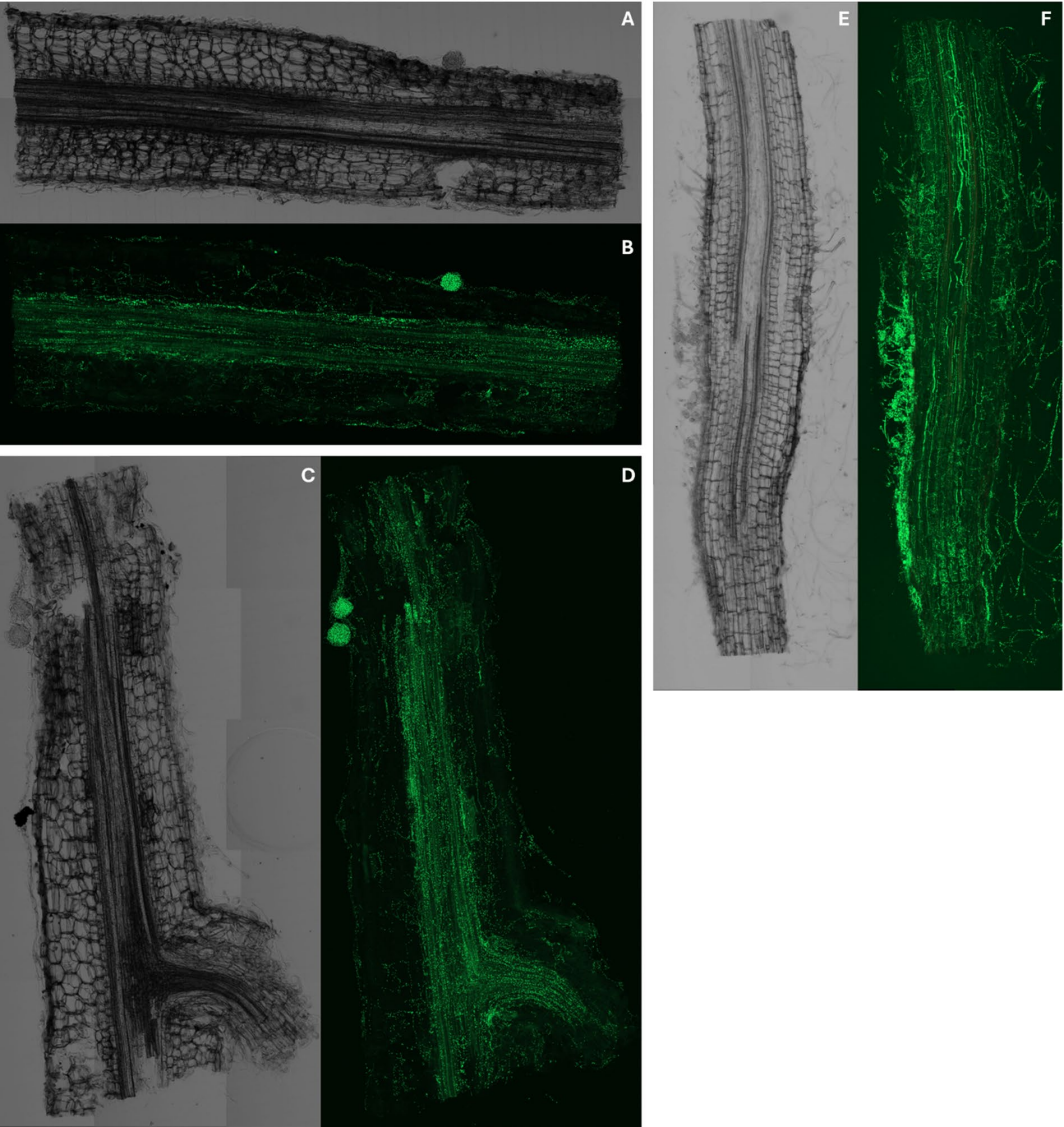
Example of the interaction between *Neurospora crassa* mutants and *Brachypodium distachyon* after 7 days with brightfield and CLSM microscopy. (**A**–**D**) Interaction of the control strain FGSC9518 with hH1::gfp fluorescence, hyphae within the root can be identified by the green fluorescent protein, which is expressed in the nuclei. These gfp signals can be seen throughout the entire root, from epidermal cells, apoplastic spaces and the vascular bundle. (**E**–**F**) Interaction of the mutant strain Δ*nce*-102, which is compatible to the control strain and showing fungal hyphae with in epidermal cells, apoplastic spaces and the vascular bundle.

During the initial four days of co-cultivation, no internal colonization was detectable via confocal or brightfield microscopy^16^. A significant transition occurred on day five, marked by the first appearance of hyphae within the outer epidermal cell layer. By day six, colonization intensified rapidly; hyphae were identified within the cortical apoplast, frequently traversing intercellular pathways. The interaction reached its maximum extent by day seven, with fungal hyphae distributed throughout the root axis. This included the vascular bundle region, where hyphae occupied intercellular spaces and were positioned adjacent to xylem elements (Fig. 1).

This kind of infection/interaction was seen in multiple mutant strains; Fig. 1 is representative for the strains Δ*nox*-2, Δ*nox*-R, Δ*so*, Δ*mak*-2, Δ*ham*-15, Δ*pla*-7, Δ*lsp*-1 and Δ*nce*-102, which all show a similar to identical strength of the interaction as well as a similar temporal pattern. Microscopic analysis for each of these strains can be found in the supplementary data. Throughout the entire infection process no necrosis, tissue collapse, or visible disease symptoms were observed in the plant at any stage.

To identify fungal factors required for the interaction, we examined a collection of *N. crassa* deletion mutant strains affecting plasma membrane organization and signaling; the collection of used strains can be seen in Table 1. To assess whether signaling pathways contribute to this interaction, *N. crassa* mutants defective in MAP kinase cascades *mak*-2 and NADPH oxidase *nox*-2 and *nox*R as well as *pla-7* and *ham-15* which take part in cell-cell communication and hyphal-fusion were tested. These five mutants exhibited colonization dynamics indistinguishable from the control strain as shown in Fig. 1. Hyphae also followed a similar temporal pattern - with the infection starting on day 5–6 after spore inoculation, and extensive colonization of the cortex and vascular tissues by day seven. This led to the assumption that classical stress- and morphogenesis-related signaling pathways might not be the key players which we were looking for in this endophytic development of *N. crassa*. Instead, the capacity to colonize plant tissues appears to depend rather on structural organization and features of the plasma membrane. These findings potentially demonstrate a mechanical distinction between the signaling-dependent pathogenesis typical of many biotrophic fungi and the structurally mediated, opportunistic colonization observed in this study.

While our set of signaling mutants produced no change in phenotype, we next explored whether eisosomal mutants might affect the interaction. We started with knock-out strains related to the eisosomal membrane complex in *Neurospora crassa*. Initial research revealed multiple genes of interest associated with the eisosomal complex such as *lsp*-1, *div*-23, *nce*-102, *ncw*-6. Knock-out mutants for most of these genes were already on stock in our lab. Further strains were generated by crossing and verified via PCR and microscopy (supplementary data). Verified strains were then used in the interaction assays and imaged with the ZEISS LSM 900.

**Fig. 2:**
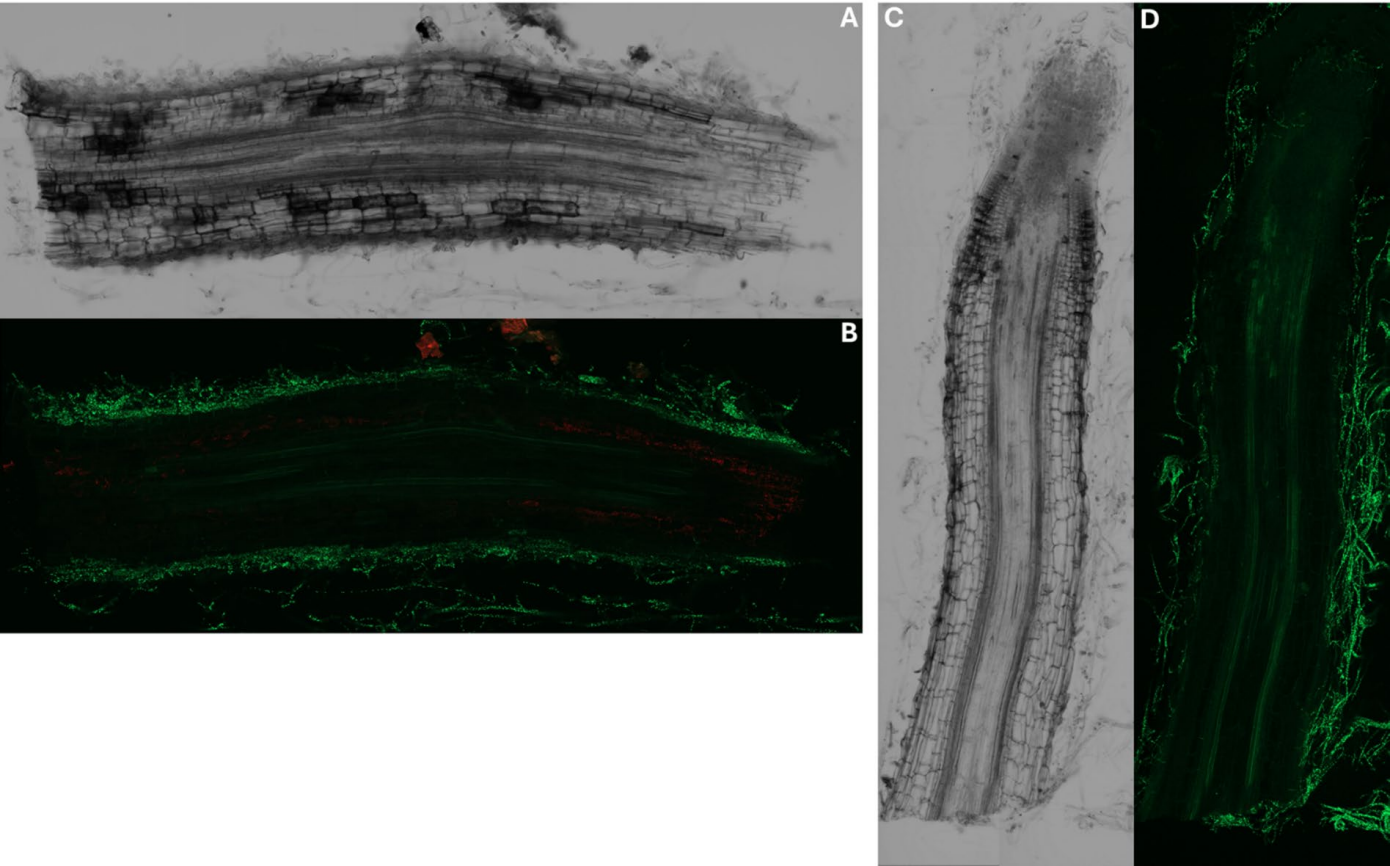
Interaction of *Neurospora crassa* mutants Δ*ncw*-6 (**A**–**B**) and Δ*div*-23 (**C**–**D**) and *Brachypodium distachyon* after 7 days of interaction with brightfield and CLSM microscopy. Fungal hyphae can be identified by the green-fluorescent protein in the nuclei via the hH1::gfp construct. The strains remain exclusively on the root surface and can’t be seen in the epidermal cells, apoplastic spaces or the vascular bundle.

Ensuing analysis revealed no significant differences during microscopy of Δ*lsp*-1 and Δ*nce*-102 compared to the control strain. However, mutants lacking *ncw*-6 or *div*-23, two proteins associated with eisosome assembly and stability, were unable to colonize *B. distachyon* roots internally (Fig. 2).

Throughout the seven-day interaction period, both Δ*ncw*-6 and Δ*div*-23 strains remained restricted to the root surface. Even upon extended co-cultivation, internal hyphae were not detectable within the epidermal or cortical tissues. Quantitative analysis of GFP-labelled hyphae confirmed a > 99% reduction in internal colonization relative to the control strain (*n* ≥ 9 roots per treatment; Fig. S1, S2). Notably, this colonization defect was not due to impaired fitness, as both mutants exhibited hyphal growth rates higher than the FGSC 9518 control.

**Fig. 3:**
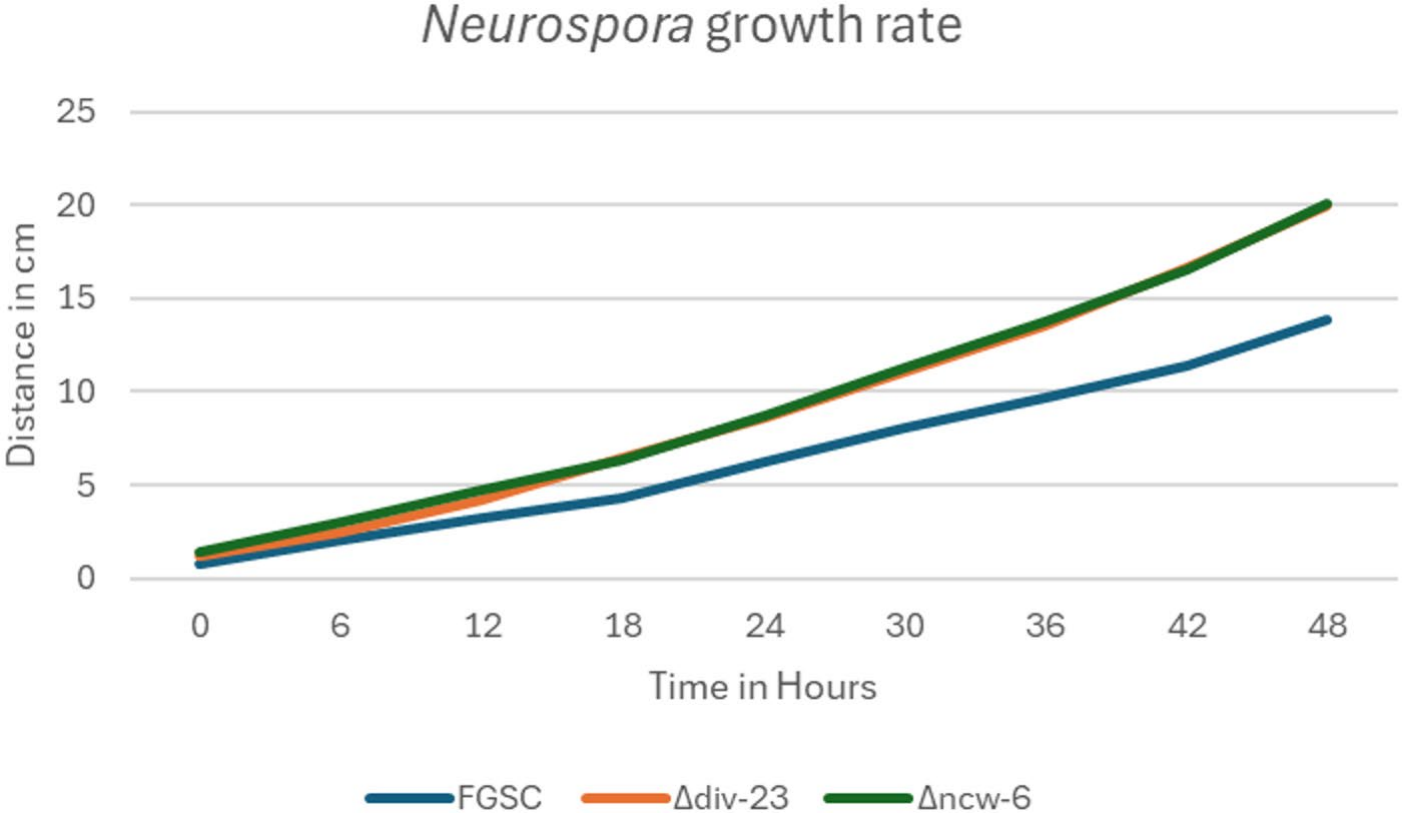
The growth of *Neurospora crassa* strains FGSC9518 (control), *Δdiv-23* and *Δncw-6* in cm per hour at 25 °C in a 40 cm tube. The strains hyphae were measured and marked every 6 h for a 48 h period.

To evaluate the general fitness of the mutant strains, linear growth rates were determined using race tube assays over a 48-hour period (Fig. 3). In this experimental set, both mutant strains exhibited significantly enhanced vegetative growth compared to the wild-type control strain FGSC9518. While the control reached an average growth rate of 0.270 cm/h (R²= 0.989), the Δdiv-23 and Δncw-6 strains grew considerably faster, recoding rates of 0.393 cm/h (R²= 0.985) and 0.384 cm/h (R²=0.984), respectively.

By the end of the 48-hour interval, the cumulative growth distance of the control strain was 13.85 ± 0.15 cm (mean ± SEM), whereas Δdiv-23 achieved a final length of 20.00 ± 0.10 cm and Δncw-6 reached 20.10 ± 0.30 cm. Analysis of variance (ANOVA) confirmed that these differences in growth rates are statistically significant (*p* < 0.001). These findings demonstrate that the previously observed defect in internal root colonization is not a consequence of impaired vegetative vigor, as the mutants effectively outperform the controls growth capacity under these conditions.

**Fig. 4:**
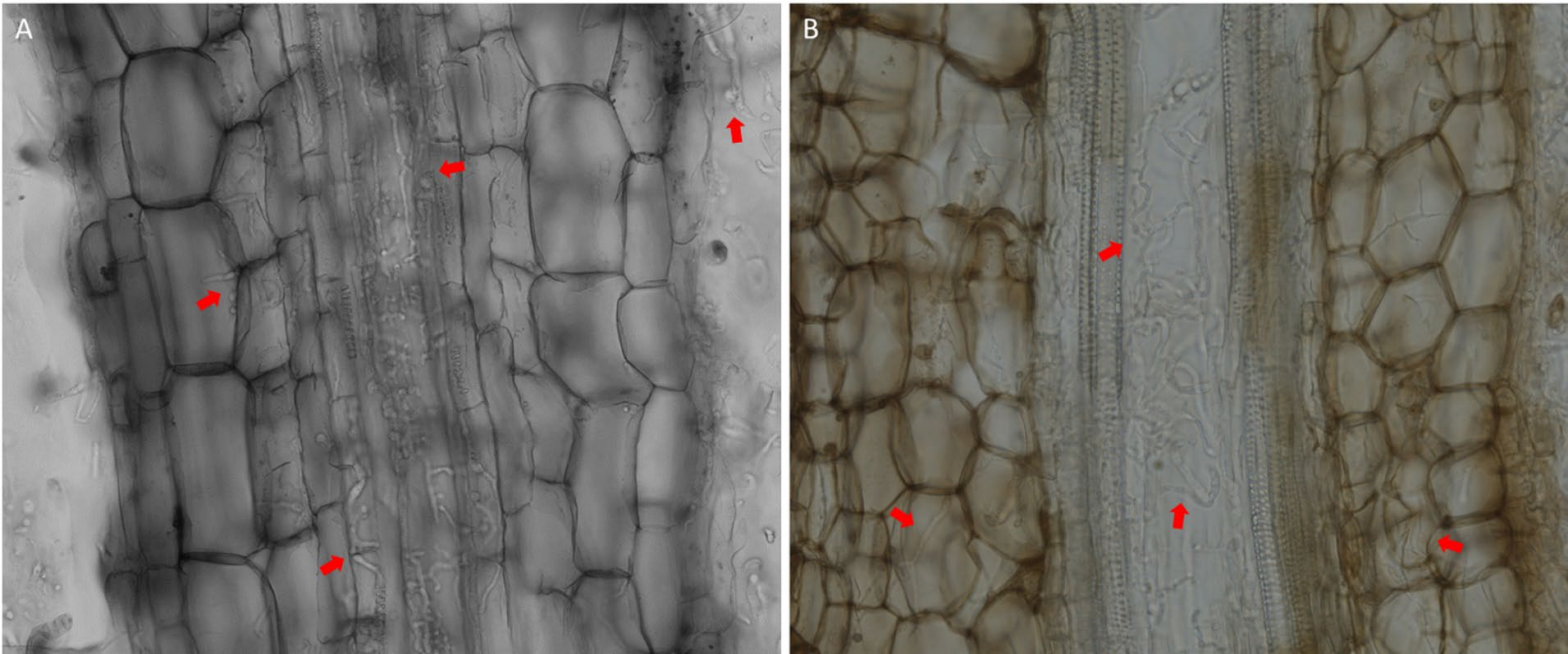
Interaction of *Neurospora crassa* mutants Δ*div*-23::*div*-23 (**A**) and Δ*ncw*-6::*ncw*-6 (**B**) with *Brachypodium distachyon* after seven days of interaction via brightfield microscopy. Fungal hyphae can be found on the outside of the root, in epidermal cells, the apoplastic spaces and the vascular bundle. Hyphae can be identified as thin, slightly brighter structures in side the root and are also marked with red arrows.

To confirm the role of the deleted genes, complementation of Δdiv-23 and Δncw-6 strains was performed using the respective wild-type alleles under the control of their native promotors (S20-S23). Microscopic analysis of these complemented strains during interaction with *B. distachyon* revealed an infection phenotype comparable to that of the wild-type control, indicating a full restoration of the colonization capacity (Fig. 4 and S3–S6).

## Discussion

*Neurospora crassa *- historically classified as a strict saprotroph - demonstrates the capacity to internally colonize *Brachypodium distachyon* roots through an opportunistic endophytic interaction. Notably, among all tested mutants, only the strains lacking the eisosome-associated proteins ncw-6 and div-23 failed to establish internal colonization. This defect occurred despite these strains displaying vegetative growth rates identical to wild-type and other signaling mutants. In contrast, mutants defective in pathogenic signaling pathways - specifically the MAP kinase Mak-2, the NADPH oxidase Nox-2, and the Soft protein - retained full colonization competence, following the same temporal progression as the control.

Eisosomes are long-lived invaginations of the fungal plasma membrane that serve as scaffolds for organizing sterol-rich domains. Their core components, including BAR-domain proteins (Lsp-1/ Pil-1) and Sur-7 family proteins, are highly conserved across the fungal kingdom^11,14^. Loss of eisosome integrity is known to disrupt membrane tension, lipid homeostasis, and cell wall organization^12,17^. Our findings echo observations in other fungal systems; for instance, in *Candida albicans*, Δsur-7 mutants exhibit disorganized membranes and severely attenuated virulence^13,14^. Similarly, in the plant pathogen *Alternaria brassicicola*, disruption of eisosome proteins reduces the formation of appressoria, thereby impairing host infection^15^.

From a structural perspective, eisosomes stabilize membrane regions exposed to environmental and host-derived stresses. Unlike specialized pathogens, *N. crassa* does not form appressoria or penetration pegs; its entry into *B. distachyon* appears purely opportunistic, likely utilizing pre-existing micro-fissures or mechanical damage. Consequently, the primary challenge for the fungus is not active penetration, but surviving the transition into the hostile apoplastic environment. This space imposes significant osmotic fluctuations, mechanical pressure, and cell wall constraints^18,19^.

We propose that the lack of functional eisosomes renders the fungal membrane less robust, compromising the resilience required to withstand these apoplastic stresses. This explains why classical signaling mutants (MAPKs, NOX), which are critical for the active infection processes of pathogens, remain fully competent in this opportunistic system. Our findings lend weight to the ‘waiting room hypothesis’^20,21^, suggesting that saprotrophic fungi maintain a dormant capacity for plant association. In *N. crassa*, eisosomes may represent a conserved structural prerequisite that facilitates this ecological plasticity, providing the mechanistic basis for fungi to explore diverse lifestyles between saprotrophy and endophytism.

## Supplementary Information

Below is the link to the electronic supplementary material.

Supplementary Material 1

## Data Availability

The datasets generated and/or used in this study are available from the corresponding author on a reasonable request.
