# Supplementary material for: Eisosomal proteins are essential for plant–fungal interaction of Neurospora crassa and the sweetgrass Brachypodium distachyon
Source: Sci Rep. 2026 Jun 9;16:17859. doi: 10.1038/s41598-026-56854-2 (PMC13250116; doi:10.1038/s41598-026-56854-2)
Supplement: Supplementary file 1 — Supplementary Material 1 [file 41598_2026_56854_MOESM1_ESM.pdf]

Supplementary

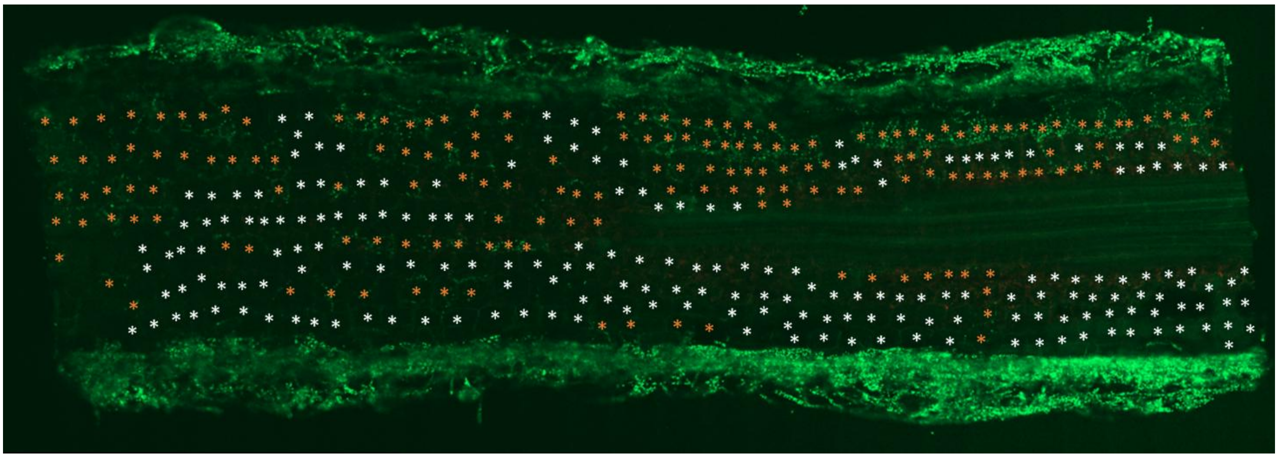

Sup. 1. Example of how the interaction between *N. crassa* and *B. distachyon* was quantified. Cut sections of the interaction were analyzed with the ZEISS900 CLSM, orange stars represent cells with in the plant root, which show infection of *N. crassa* hyphae. These can be identified by the green dots via the H1::gfp fluoresces. Cells without certain infection in form of the gfp-signal were marked with white stars. Stars were counted an put into a graph, counting infected and non-infected cells is a common practice trying to quantify mycorrhizal interactions.

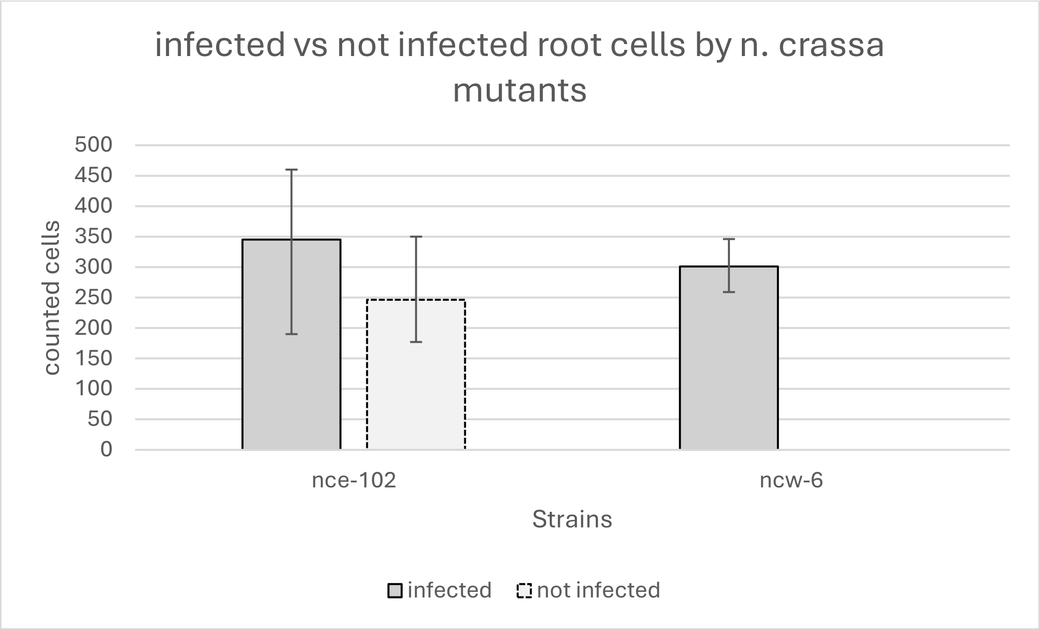

Sup. 2. This graphic shows the interaction of two *N. crassa* strains Δnce-102 and Δncw-6 based on counted infected and non-infected plant root cells, like seen in Sup. 1.

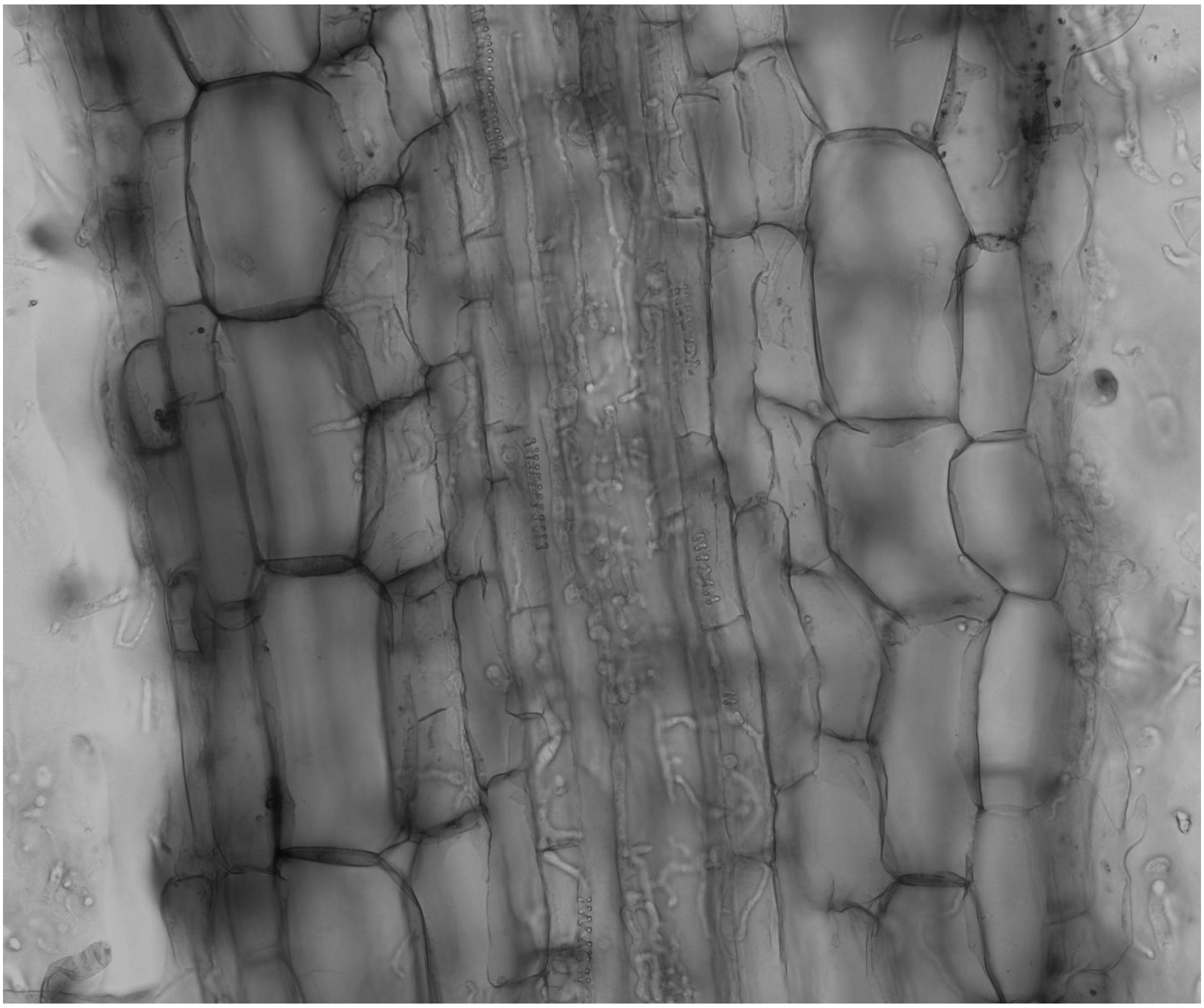

Sup. 3. Brightfield microscopy of *Brachypodium distachyon* and *Neurospora crassa* co-cultivation. This shows the complementation strain Δdiv23::div23, hyphae of the fungus can be seen in the middle within the vascular bundle as well as on the outside of the root.

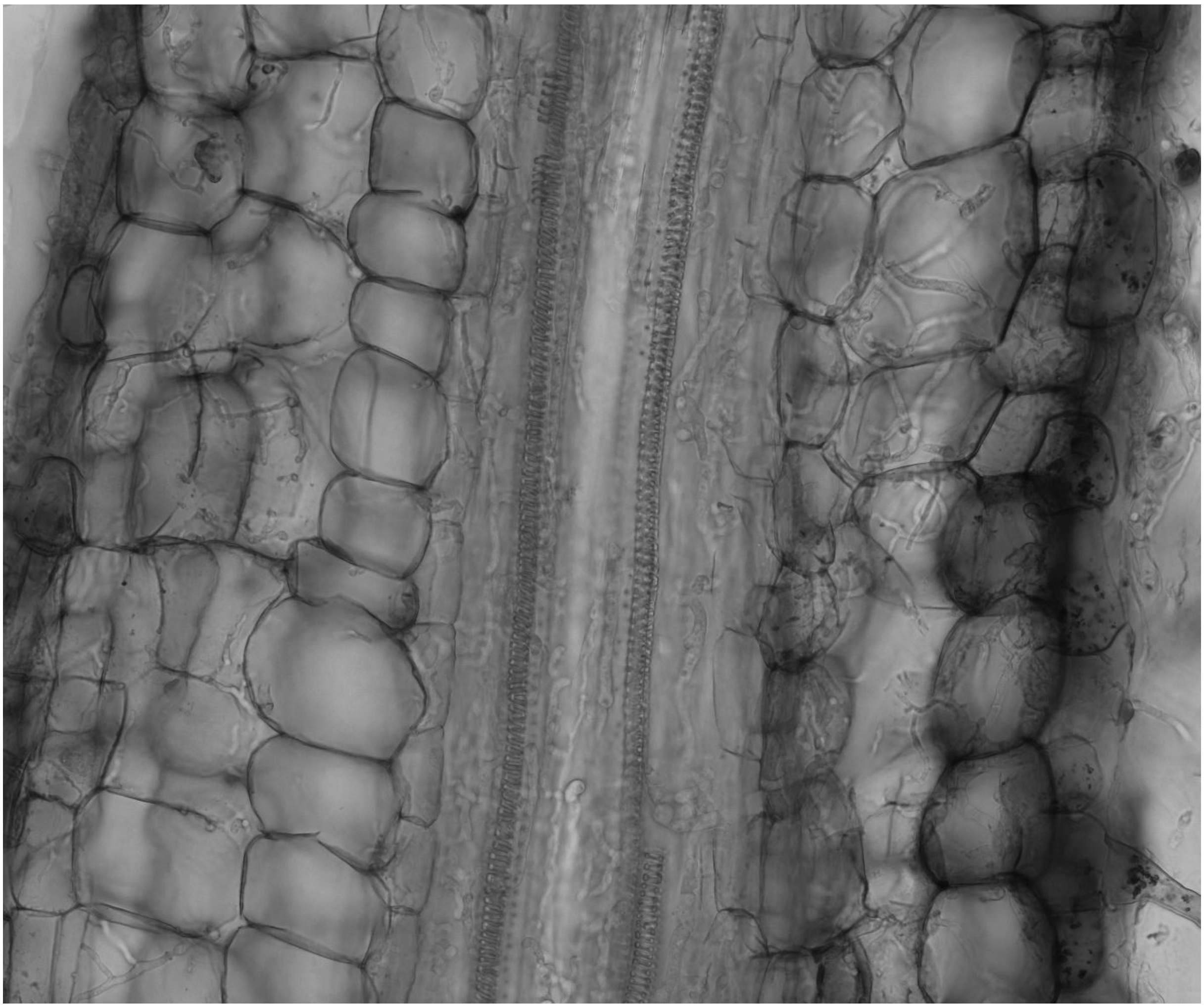

Sup. 4. Brightfield microscopy of *Brachypodium distachyon* and *Neurospora crassa* co-cultivation. This shows the complementation strain Δdiv23::div23 - hyphae of the fungus can be seen in the middle within the vascular bundle, epidermal cells and on the outside of the root.

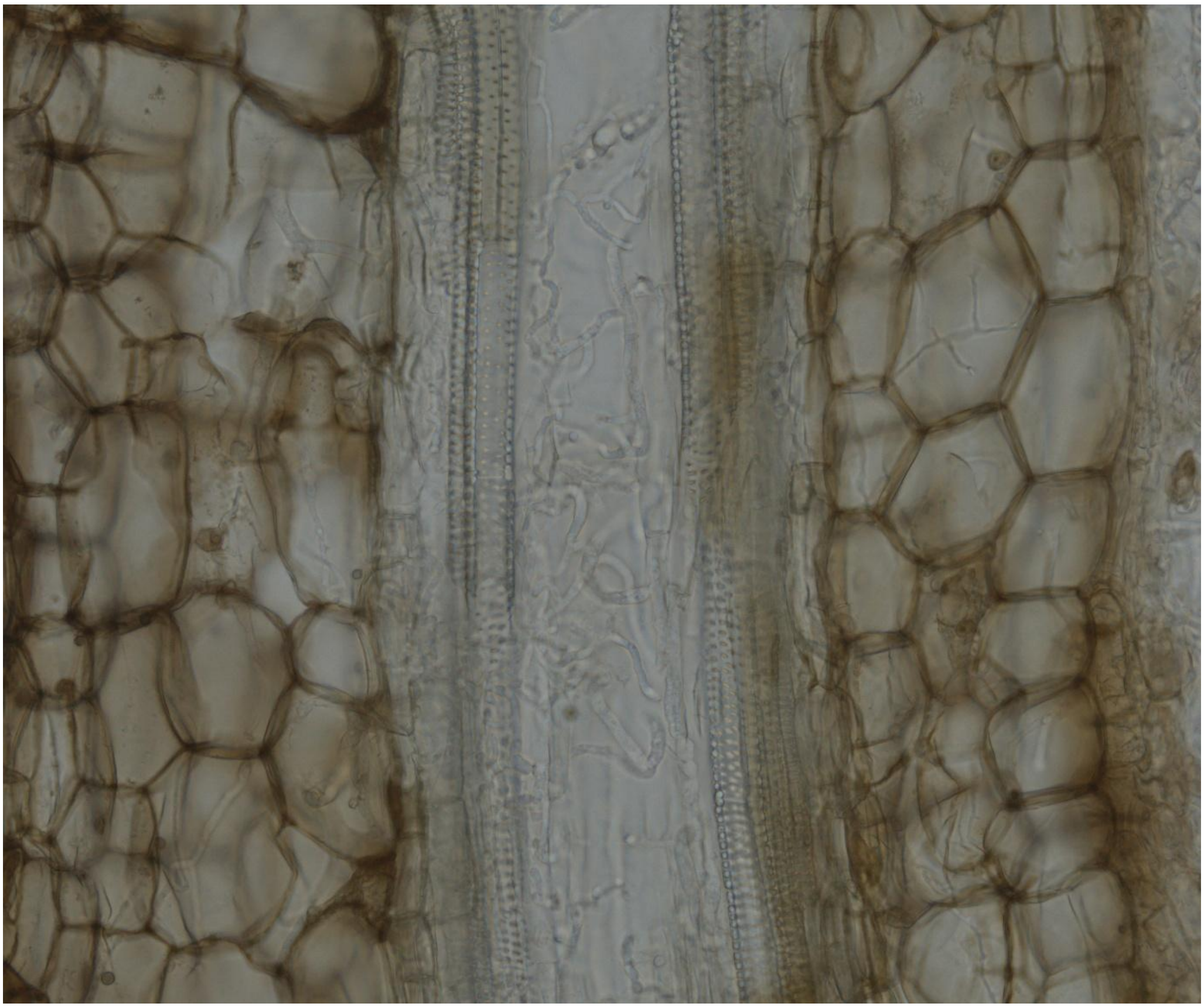

Sup. 5. Brightfield microscopy of *Brachypodium distachyon* and *Neurospora crassa* co-cultivation. This shows the complementation strain Δncw6::ncw6 - hyphae of the fungus can be seen in the middle within the vascular bundle, epidermal cells and on the outside of the root.

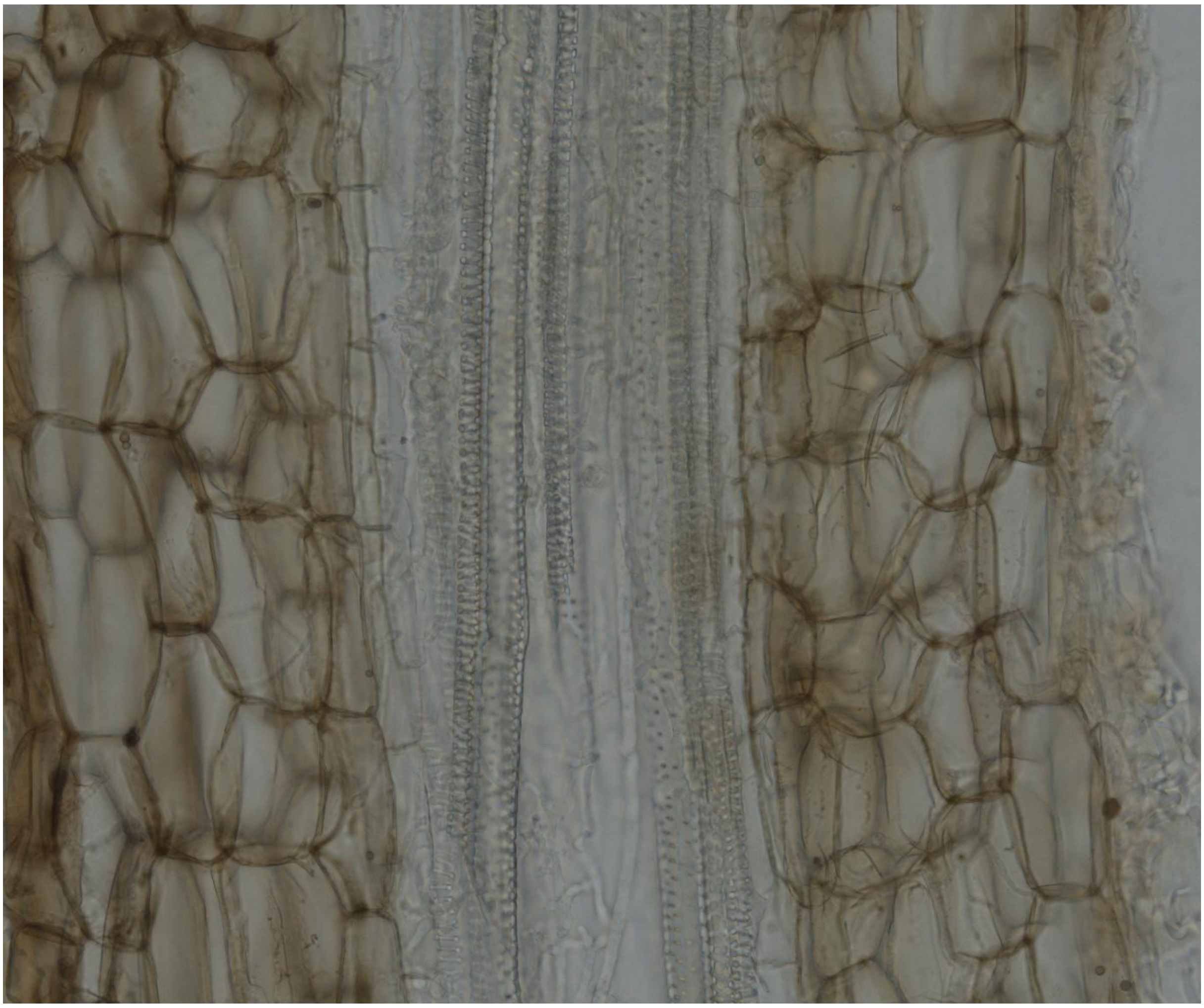

Sup. 6. Brightfield microscopy of *Brachypodium distachyon* and *Neurospora crassa* co-cultivation. This shows the complementation strain Δncw6::ncw6 - hyphae of the fungus can be seen in the middle within the vascular bundle, epidermal cells and on the outside of the root.

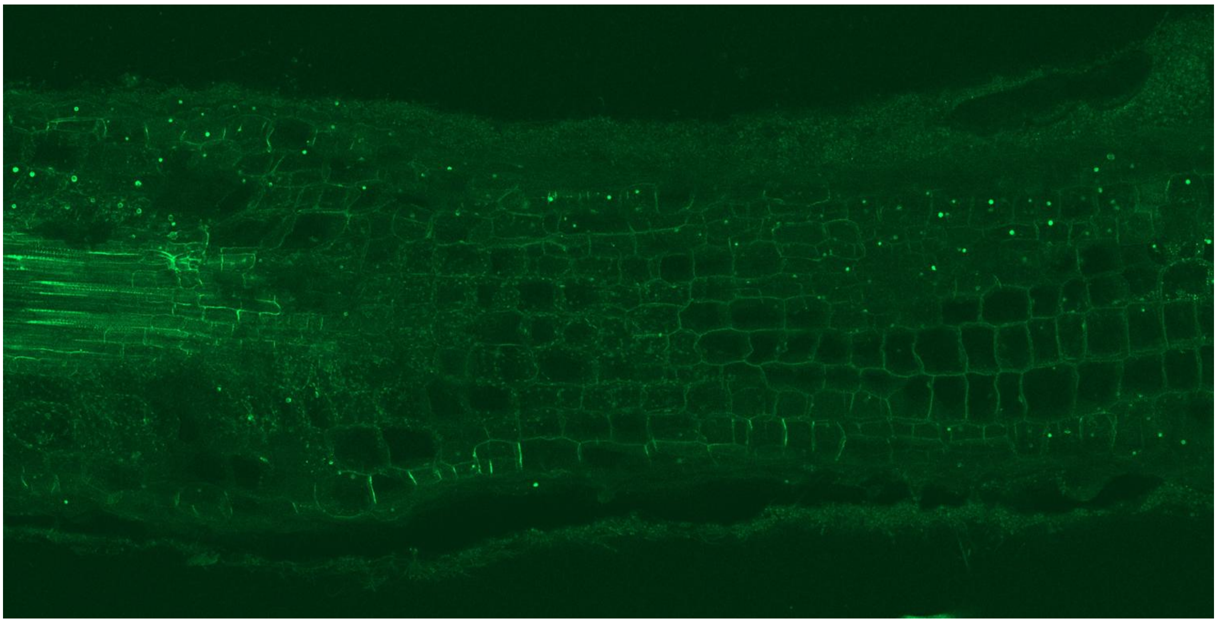

Sup. 7. Fluorescent microscopy of *Brachypodim distachyon* and *Neurospora crassa* co-cultivation. This shows the interaction of the Δso mutant - hyphae of the fungus can be seen in the

middle within the vascular bundle, epidermal cells and on the outside of the root. The green fluorescent of the hH1::gfp is not as strong as in other cases since the Δso phenotype expresses small and thin hyphae.

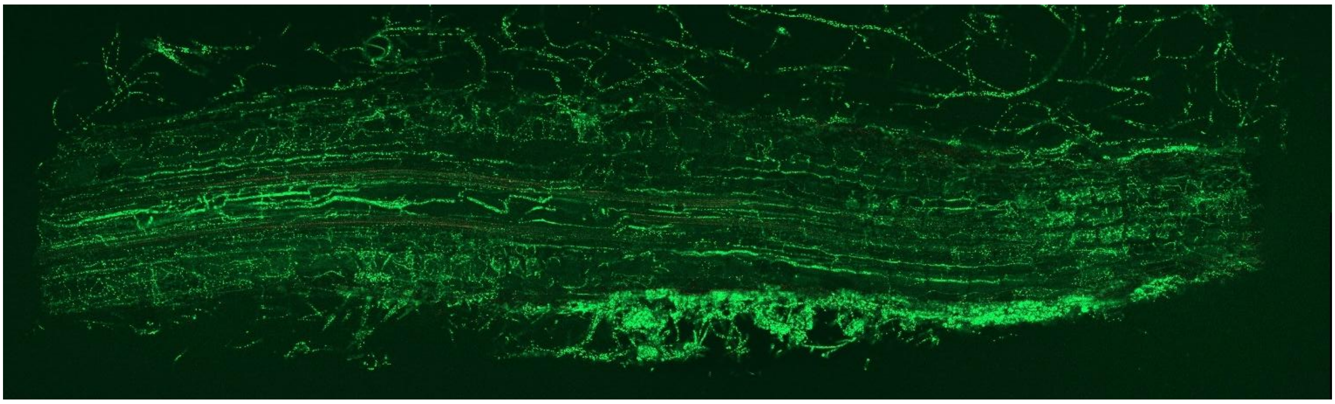

Sup. 8. Fluorescent microscopy of *Brachypodium distachyon* and *Neurospora crassa* co-cultivation. This shows the interaction of the Δnce102 mutant - hyphae of the fungus can be seen in the middle within the vascular bundle, epidermal cells and on the outside of the root. The green fluorescent is due to the hH1::gfp construct.

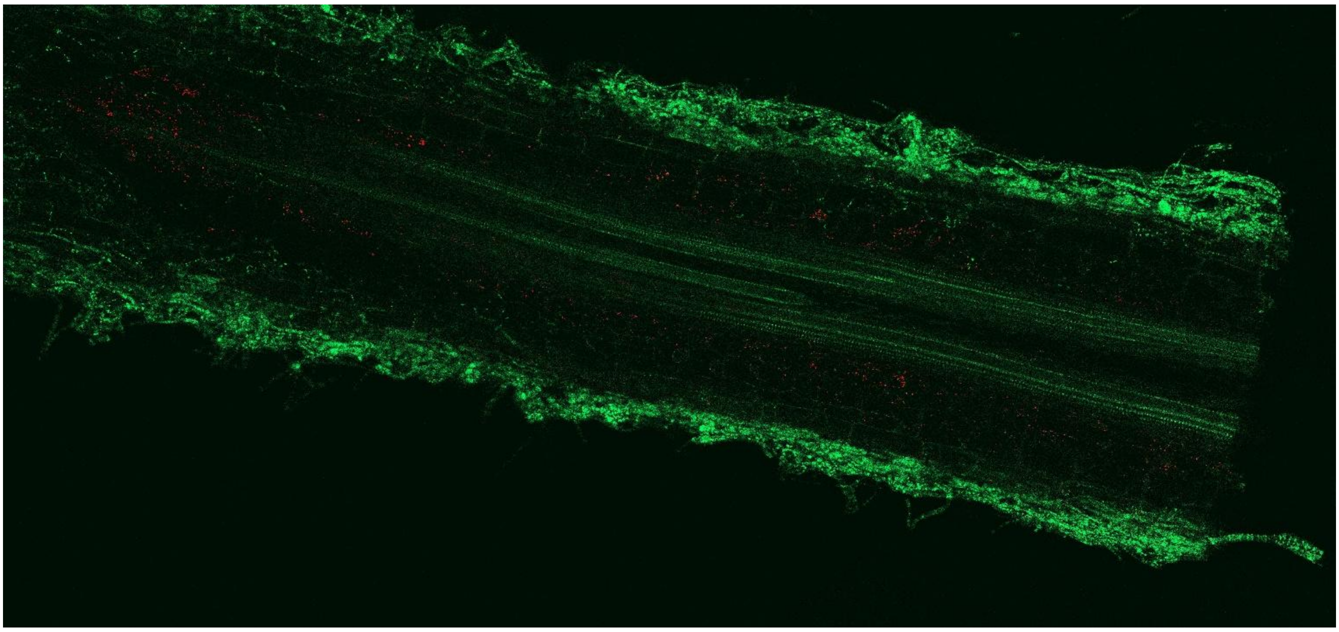

Sup. 9. Fluorescent microscopy of *Brachypodium distachyon* and *Neurospora crassa* co-cultivation. This shows the interaction of the Δlsp1 mutant - hyphae of the fungus can be seen in the middle within the vascular bundle, epidermal cells and on the outside of the root. The green fluorescent is due to the hH1::gfp construct.

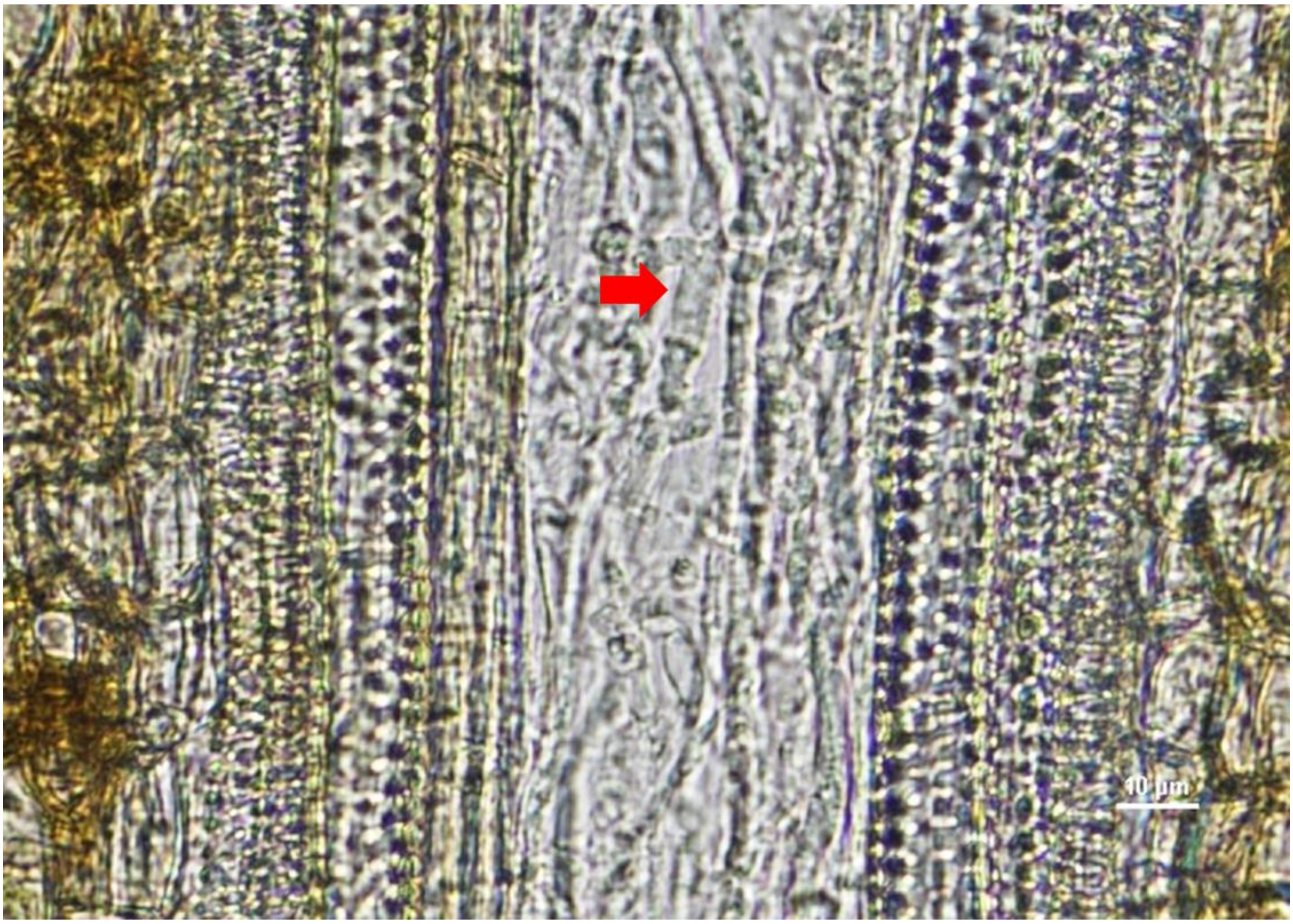

Sup. 10. Brightfield microscopy of *Brachypodium distachyon* and *Neurospora crassa* co-cultivation. This shows the interaction of the Δham-15 mutant - hyphae of the fungus can be seen in the middle within the vascular bundle.

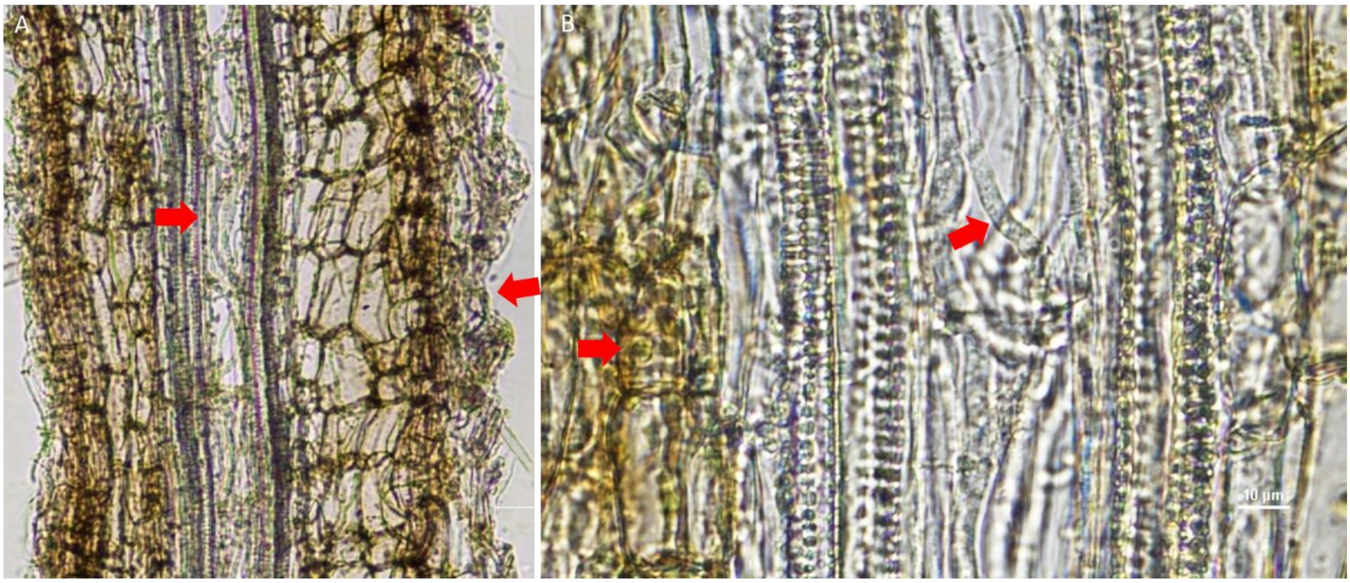

Sup. 11. Brightfield microscopy of *Brachypodium distachyon* and *Neurospora crassa* co-cultivation. This shows the interaction of the Δnox-2 mutant - hyphae of the fungus can be seen in the middle within the vascular bundle (A-B) and on the outside of the root, as well as in epidermal cells (A-B).

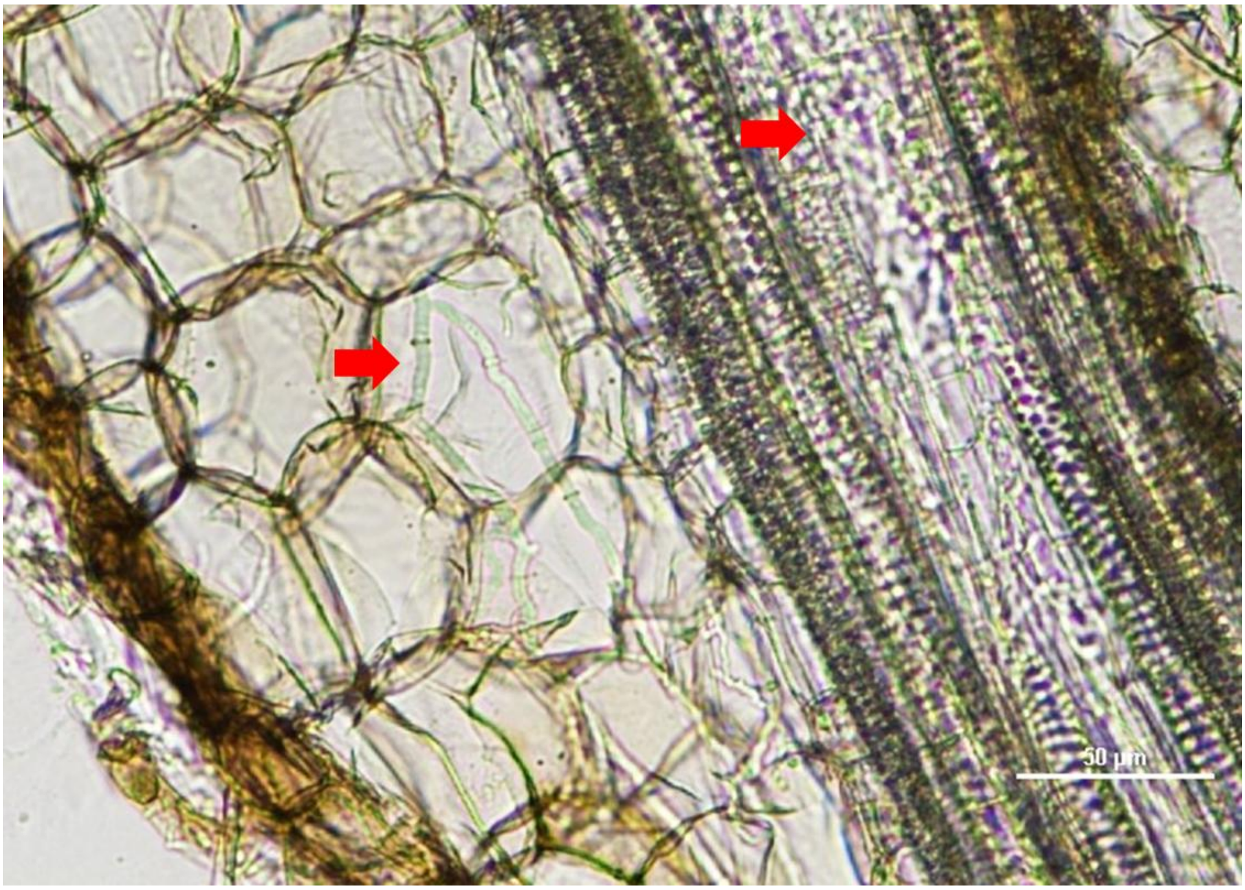

Sup. 12. Brightfield microscopy of *Brachypodium distachyon* and *Neurospora crassa* co-cultivation. This shows the interaction of the ΔnoxR mutant - hyphae of the fungus can be seen in the middle within the vascular bundle and epidermal cells.

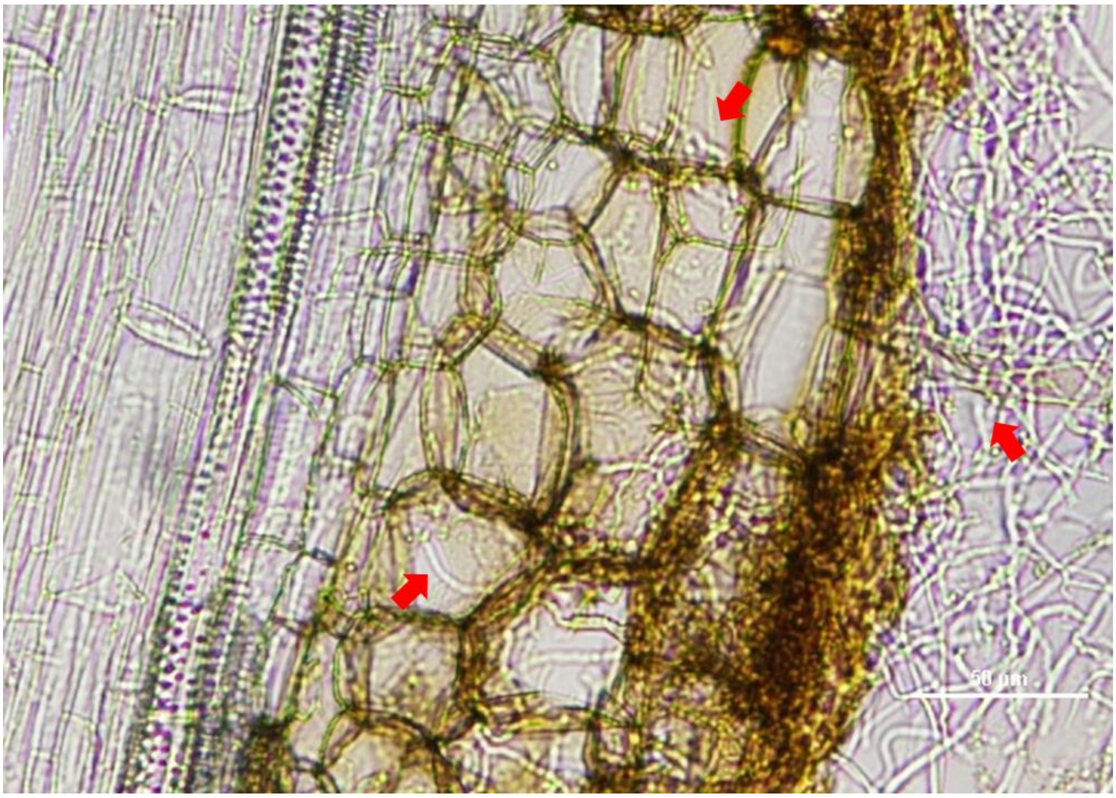

Sup. 13. Brightfield microscopy of *Brachypodium distachyon* and *Neurospora crassa* co-cultivation. This shows the interaction of the Δmak-2 mutant - hyphae of the fungus can be seen in epidermal cells and on the outside of the root.

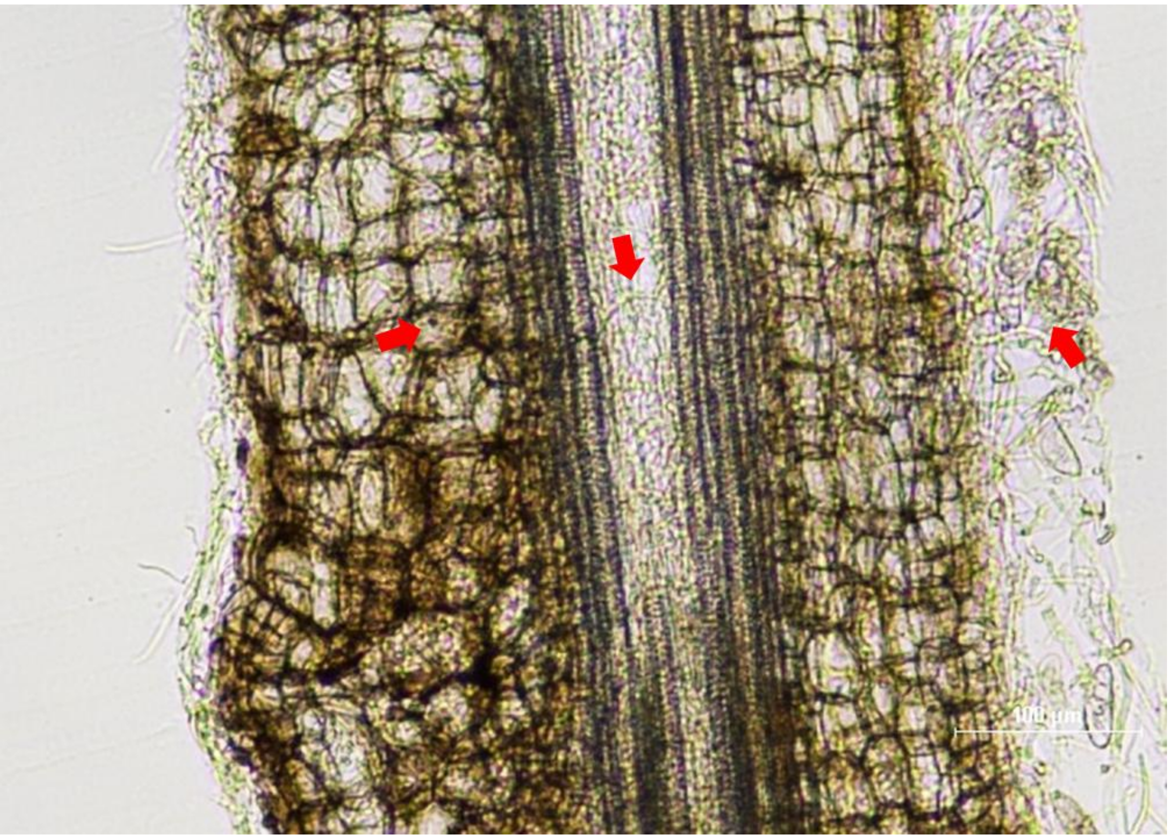

Sup. 14. Brightfield microscopy of *Brachypodium distachyon* and *Neurospora crassa* co-cultivation. This shows the interaction of the Δpla-7 mutant - hyphae of the fungus can be seen in the middle within the vascular bundle, epidermal cells and the outside of the root.

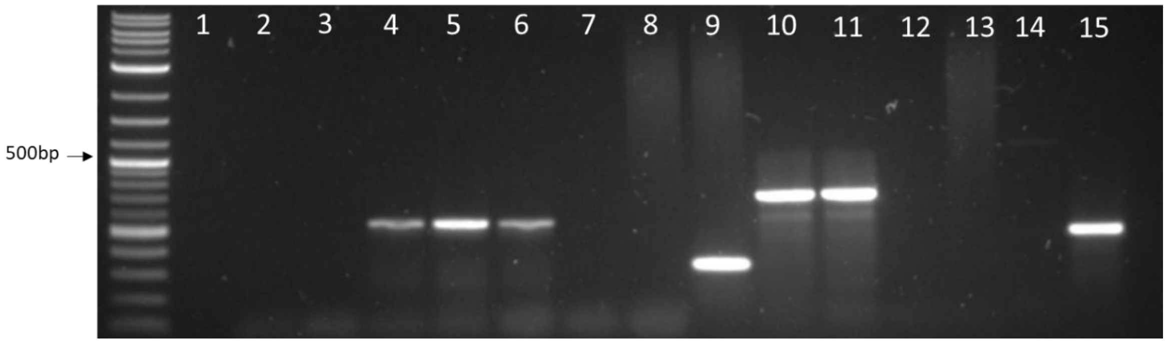

Sup. 15. Gel electrophoresis of control PCR of Δdiv-23 and Δlsp-1. Lines 1-3 primers binding to the div-23 sequence were used; no bands indicating the div-23 DNA could not be amplified. Lines 4-6 primers binding to the his-cassette of the div-23 knock-out were used, bands indicating DNA of the his-cassette was amplified, hence showing a successful knockout strain. Line 7-9 primer for the lsp-1 sequence were used; while in 7 and 8 no lsp-1 specific DNA was amplified in line 9 DNA was amplified. Lines 10-12 primers binding to the his-cassette of the lsp-1 knock-out were used; 10-11 showing the amplification of the his-cassette indicating the knock-out strain, line 12 shows no band indicating no knock-out. Lines 13-15 showing H2O and WT controls.

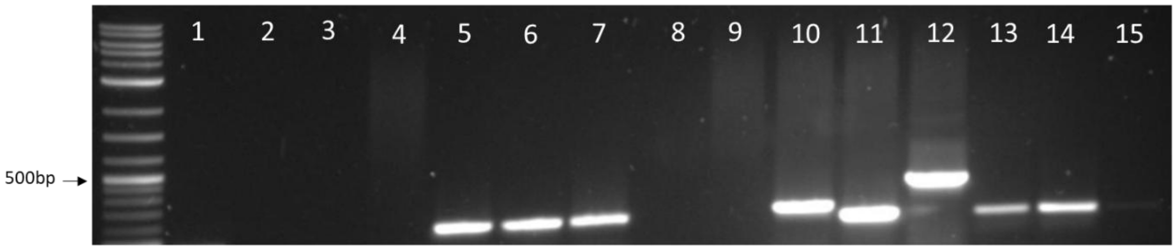

Sup. 16. Gel electrophoresis of control PCR of Δncw-6 and Δnce-102. Lines 2-4 primers binding to the ncw-6 gene were used; no bands indicating no ncw-6 DNA was amplified. Lines 5-7 primers binding to the his.cassette of the ncw-6 knock-out were used, bands indicating DNA of the his-cassette was amplified, therefore showing a knock-out strain. Lines 8 and 9 primers binding to the nce-102 gene were used; no bands indicating no nce-102 DNA was amplified. Lines 10 and 11 primers binding to the his-cassette of the nce-102 knock-out were used; bands indicating the amplification of the his-cassette. Lines 12-15 showing WT and H2O controls.

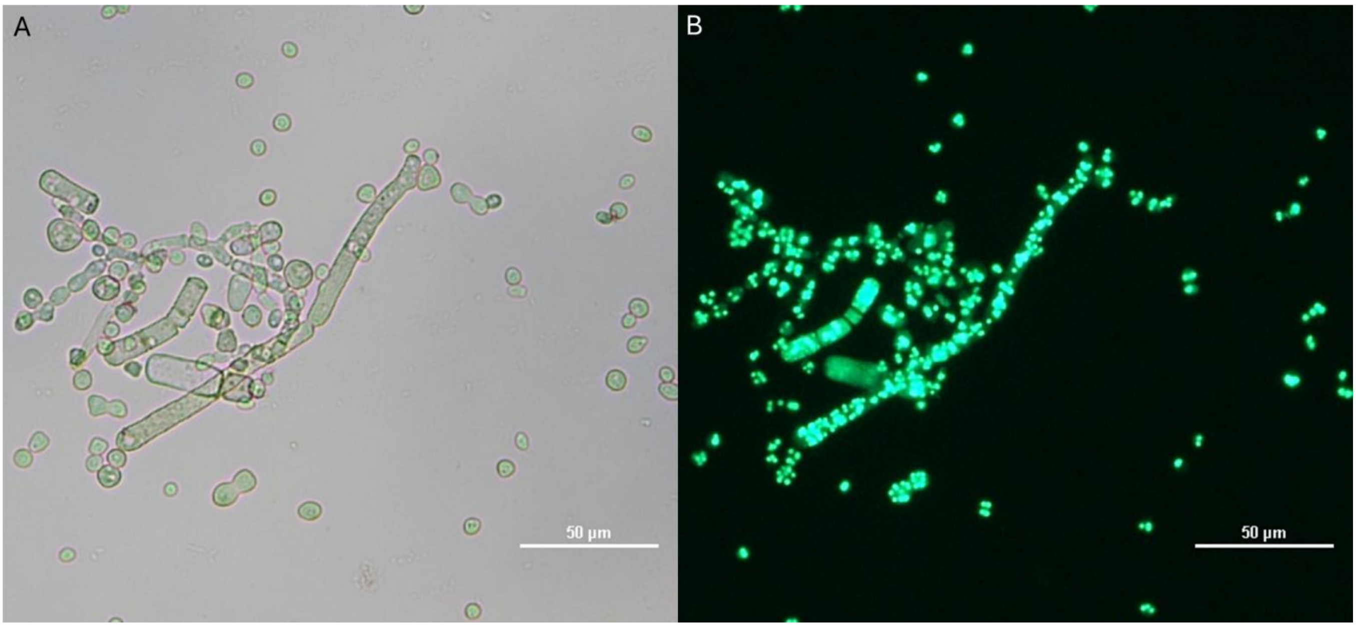

Sup. 17. Brightfield and fluorescent microscopy of the *Neurospora crassa* strain FGSC9518. This strain strain was used in co-cultivation assays with *Brachypodium distachyon* and showing hH1::gfp fluoresces of the green-fluorescent-protein in the nuclei of the fungal hyphae and conidia. FGSC9518 was used as the control strain in the co-cultivation assays.

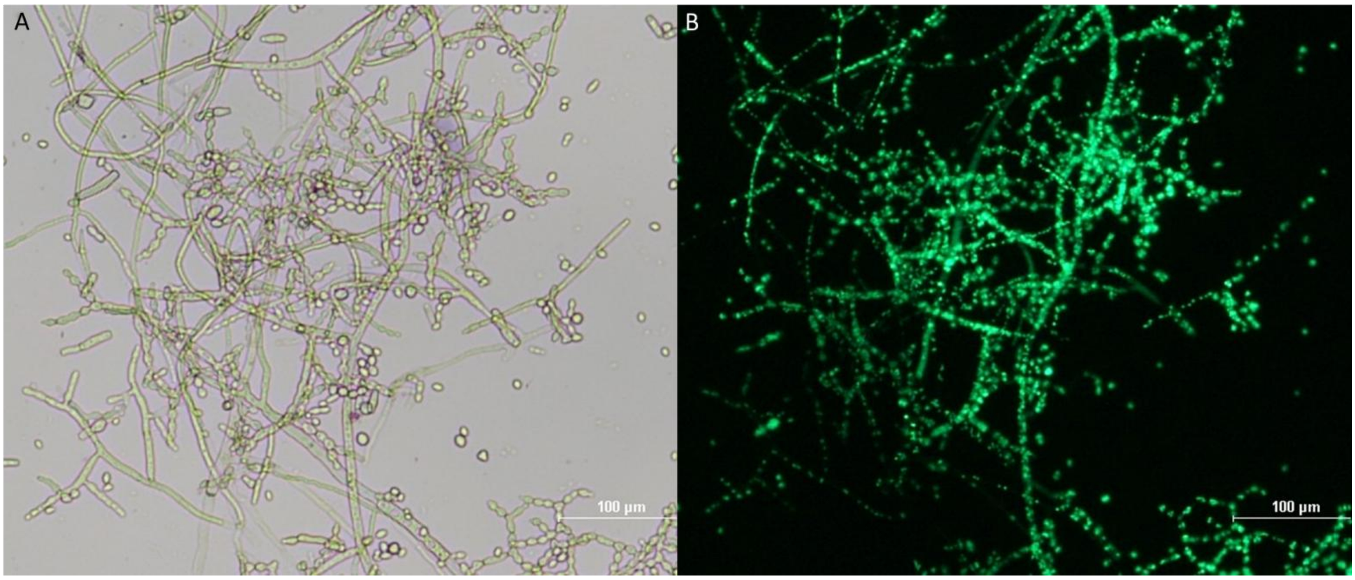

Sup. 18. Brightfield and fluorescent microscopy of the *Neurospora crassa* mutant Δncw6::hH1gfp. This mutant strain was used in co-cultivation assays with *Brachypodium distachyon* and showing hH1::gfp fluoresces of the green-fluorescent-protein in the nuclei of the fungal hyphae and conidia.

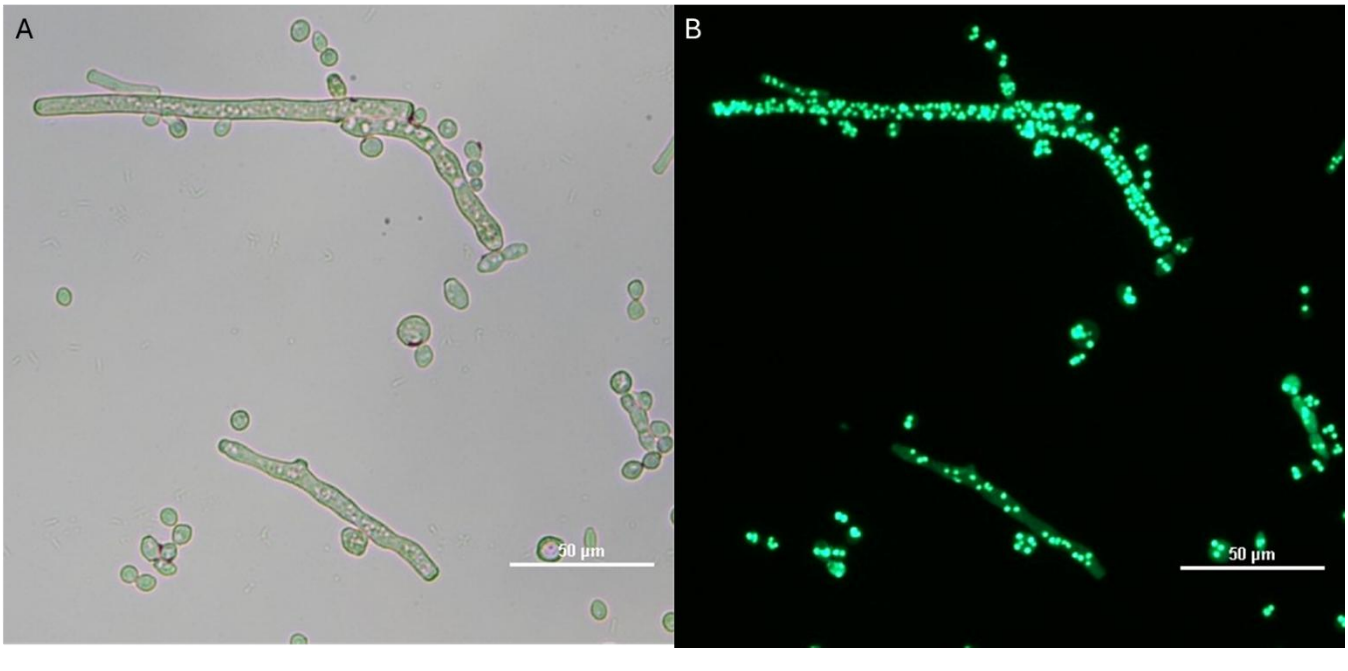

Sup. 19. Brightfield and fluorescent microscopy of the *Neurospora crassa* mutant Δdiv23::hH1gfp. This mutant strain was used in co-cultivation assays with *Brachypodium distachyon* and showing hH1::gfp fluoresces of the green-fluorescent-protein in the nuclei of the fungal hyphae and conidia.

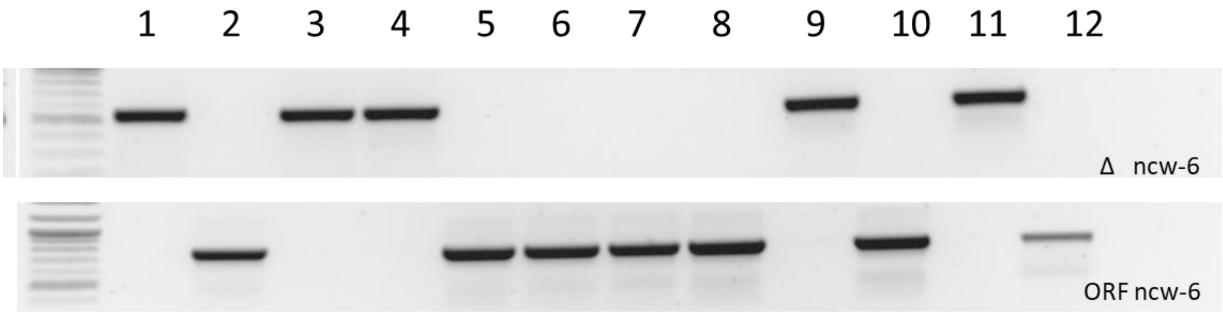

Sup. 20. Gel electrophoresis for PCR control for the *Neurospora crassa* knockout strain *Δncw6* in the his3- background. Two PCRs were done to confirm the strain; first with primers binding in the his-cassette of the removed gene and second primers binding on the genes sequence. Hence no band on the lower row indicating the gene is not present in the isolated DNA and a band present in the upper row indicating the presence of the his-cassette which was introduced in the gene's region by the fungal genetic stock center. Line 1 to 10 are the gDNA isolates of experiment AS098_1 – AS098_10, 11 represents the original *Δncw6* strain FGSC16722 as positive control and 12 is FGSC9716 as a negative control.

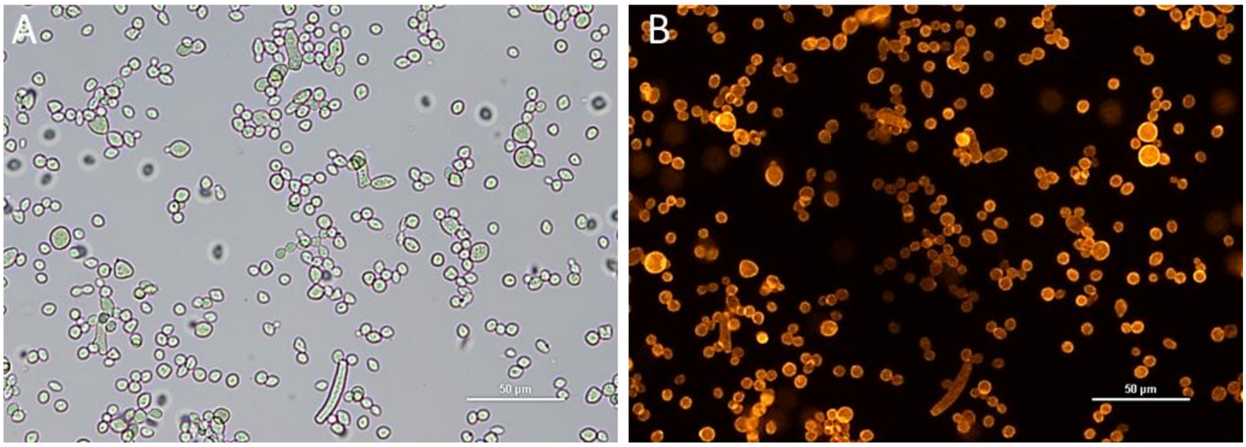

Sup. 21. Brightfield and fluorescens microscopy of the complementation strain *Δncw6::ncw6rfp*. Complementation of the knockout was accomplished by transformation of the plasmid pAS930 and confirmed by expression of rfp signals.

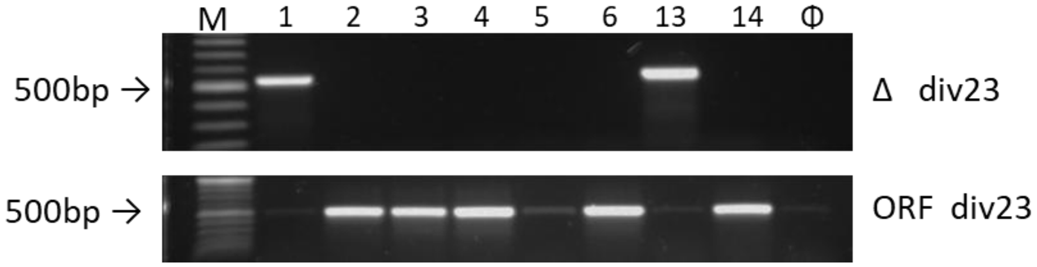

Sup. 22. Gel electrophoresis for PCR control for the *Neurospora crassa* knockout strain *Δdiv23* in the his3- background. Two PCRs were done to confirm the strain; first with primers binding in the

his-cassette of the removed gene and second primers binding on the genes sequence. Hence no band on the lower row indicating the gene is not present in the isolated DNA and a band present in the upper row indicating the presence of the his-cassette which was introduced in the gene's region by the fungal genetic stock center. Line 1 representing the knockout strain in the his3- background; line 13 the positive control with the FGSC14455 strain; 2, 3, 4, 5, 6 showing no successful knockout of *Δdiv23* in the his3- background. Line 14 represents the negative control and the last line the H2O control.

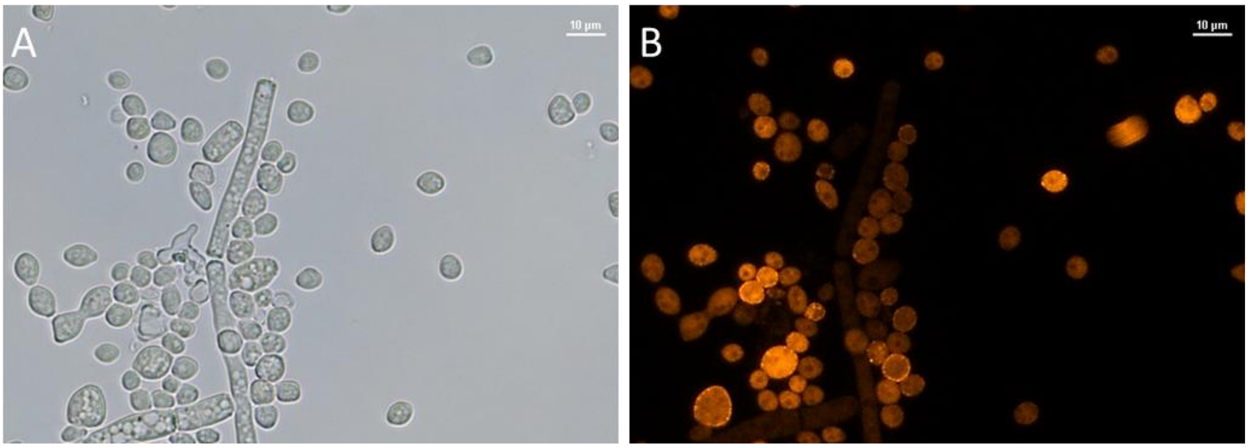

Sup. 23. Brightfield and fluorescens microscopy of the complementation strain *Δdiv23::div23rfp*. Complementation of the knockout was accomplished by transformation of the plasmid pAS925 and confirmed by expression of rfp signals.
